# Structural basis of a shared antibody response to SARS-CoV-2

**DOI:** 10.1126/science.abd2321

**Published:** 2020-07-13

**Authors:** Meng Yuan, Hejun Liu, Nicholas C. Wu, Chang-Chun D. Lee, Xueyong Zhu, Fangzhu Zhao, Deli Huang, Wenli Yu, Yuanzi Hua, Henry Tien, Thomas F. Rogers, Elise Landais, Devin Sok, Joseph G. Jardine, Dennis R. Burton, Ian A. Wilson

**Affiliations:** 1 Department of Integrative Structural and Computational Biology, The Scripps Research Institute, La Jolla, CA 92037, USA.; 2 Department of Immunology and Microbiology, The Scripps Research Institute, La Jolla, CA 92037, USA.; 3 IAVI Neutralizing Antibody Center, The Scripps Research Institute, La Jolla, CA 92037, USA.; 4 Consortium for HIV/AIDS Vaccine Development (CHAVD), The Scripps Research Institute, La Jolla, CA 92037, USA.; 5 Division of Infectious Diseases, Department of Medicine, University of California, San Diego, La Jolla, CA 92037, USA.; 6 IAVI, New York, NY 10004, USA.; 7 Ragon Institute of Massachusetts General Hospital, Massachusetts Institute of Technology, and Harvard University, Cambridge, MA 02139, USA.; 8 The Skaggs Institute for Chemical Biology, The Scripps Research Institute, La Jolla, CA, 92037, USA.

## Abstract

Molecular understanding of neutralizing antibody responses to severe acute respiratory syndrome coronavirus 2 (SARS-CoV-2) could accelerate vaccine design and drug discovery. We analyzed 294 anti–SARS-CoV-2 antibodies and found that immunoglobulin G heavy-chain variable region 3-53 (IGHV3-53) is the most frequently used IGHV gene for targeting the receptor-binding domain (RBD) of the spike protein. Co-crystal structures of two IGHV3-53–neutralizing antibodies with RBD, with or without Fab CR3022, at 2.33- to 3.20-angstrom resolution revealed that the germline-encoded residues dominate recognition of the angiotensin I converting enzyme 2 (ACE2)–binding site. This binding mode limits the IGHV3-53 antibodies to short complementarity-determining region H3 loops but accommodates light-chain diversity. These IGHV3-53 antibodies show minimal affinity maturation and high potency, which is promising for vaccine design. Knowledge of these structural motifs and binding mode should facilitate the design of antigens that elicit this type of neutralizing response.

## A common theme in antibody responses

In the fight against severe acute respiratory syndrome coronavirus 2 (SARS-CoV-2), antibodies are a key tool, both as potential therapeutics and to guide vaccine development. Yuan *et al.* focused on finding shared antibody responses, in which multiple individuals develop antibodies against the same antigen using the same genetic elements and modes of recognition. The authors identified the immunoglobulin heavy-chain variable region 3-53 gene as the most frequently used among 294 antibodies that target the receptor-binding domain (RBD) of the viral spike protein. These antibodies have few somatic mutations, and crystal structures of two neutralizing antibodies bound to the RBD show that mostly germline-encoded residues are involved in binding. The minimal affinity maturation and high potency of these antibodies is promising for vaccine design.

*Science*, this issue p. 1119

## Teaser

IGHV3-53–encoded antibodies that display high-affinity germline binding to SARS-CoV-2 are a common response to COVID-19.

The ongoing coronavirus disease 2019 (COVID-19) pandemic caused by severe acute respiratory syndrome coronavirus 2 (SARS-CoV-2) has resulted in enormous global health and socioeconomic damage and requires urgent development of an effective vaccine (*1*). Although multiple vaccine candidates have entered clinical trials (*2*), the molecular features that contribute to an effective antibody response are not clear. Shared antibody responses to specific microbial pathogens have been found in which the same genetic elements and modes of recognition are observed in multiple individuals against a given antigen. Such responses to microbial pathogens have been observed against influenza (*3*), dengue (*4*), malaria (*5*), and HIV (*6*). Characterization of the molecular interactions between pathogens and cognate antigen can provide insight into how the immune repertoire is able to quickly respond to novel microbial pathogens and will facilitate the rational design of vaccines against them (*7*, *8*).

**Fig. 1:**
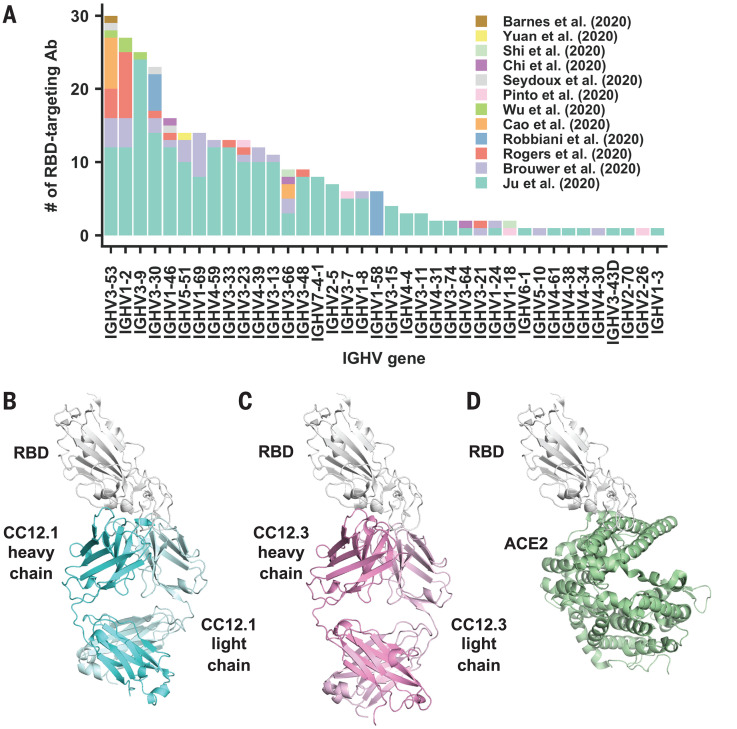
Structures of two IGHV3-53 antibodies. (**A**) The distribution of IGHV gene usage is shown for a total of 294 RBD-targeting antibodies (*17*–*28*). (**B** to **D**) Crystal structures of (B) CC12.1 in complex with SARS-CoV-2 RBD, (C) CC12.3 with SARS-CoV-2 RBD, and (D) human ACE2 with SARS-CoV-2 RBD (PDB 6M0J) (*12*).

The spike (S) protein is the major surface antigen of SARS-CoV-2. The S protein uses its receptor-binding domain (RBD) to engage the host receptor angiotensin I converting enzyme 2 (ACE2) for viral entry (*9*–*12*). RBD-targeting antibodies could then neutralize SARS-CoV-2 by blocking ACE2 binding. A number of antibodies that target the RBD of SARS-CoV-2 have now been discovered (*13*–*28*). We compiled a list of 294 SARS-CoV-2 RBD-targeting antibodies for which information on immunoglobulin G heavy-chain variable (IGHV) gene usage is available (*17*–*28*) (table S1) and found that IGHV3-53 is the most frequently used IGHV gene among these antibodies (Fig. 1A), with 10% encoded by IGHV3-53, compared with 0.5 to 2.6% (mean 1.8%) in the repertoire of naïve healthy individuals (*29*, *30*). IGHV3-53 antibodies were found in seven of 12 studies and in 17 of 32 COVID-19 patient samples (*17*–*28*, *31*). These IGHV3-53 antibodies not only had lower somatic mutation rates but also were more potent compared with other germ lines in the cohort investigated here (*27*) (fig. S1). The prevalence of IGHV3-53 in the antibody response in SARS-CoV-2 patients has also been recognized in other antibody studies (*20*, *22*, *27*).

To understand the molecular features that endow IGHV3-53 with favorable properties for RBD recognition, we determined the crystal structures of two IGHV3-53–neutralizing antibodies, CC12.1 and CC12.3, in complex with the SARS-CoV-2 RBD and with the cross-reactive Fab CR3022 to SARS-like CoVs (*17*). CC12.1 and CC12.3 were previously isolated from a SARS-CoV-2–infected patient and were shown to be specific for the RBD (*27*). CC12.1 and CC12.3 [median inhibitory concentration (IC_50_), ~20 ng/ml] were among the top four highly potent neutralizing antibodies in the panel of antibodies assayed against live replicating SARS-CoV-2 virus and pseudovirus (*27*). Although CC12.1 and CC12.3 are both encoded by IGHV3-53, CC12.1 uses IGHJ6, IGKV1-9, and IGKJ3, whereas CC12.3 uses IGHJ4, IGKV3-20, and IGKJ1. This variation in IGHJ, IGKV, and IGKJ usage indicates that CC12.1 and CC12.3 belong to different clonotypes but are encoded by a common IGHV3-53 germline gene (fig. S2). IgBlast analysis (*32*) showed that IGHV and IGKV of CC12.1 have acquired only four amino acid changes (somatic mutations) during affinity maturation from the original germline antibody sequence (fig. S2, A and B). Similarly, CC12.3 is also minimally somatically mutated, with three amino acid changes in IGHV and a single amino acid deletion in IGKV (fig. S2, A and C). The binding affinities (*K*_d_) of the Fabs CC12.1 and CC12.3 to SARS-CoV-2 RBD are 17 and 14 nM, respectively (fig. S3). Moreover, competition experiments suggest that CC12.1 and CC12.3 bind to a similar epitope, which overlaps with the ACE2-binding site but not the CR3022 epitope (fig. S4).

**Fig. 2:**
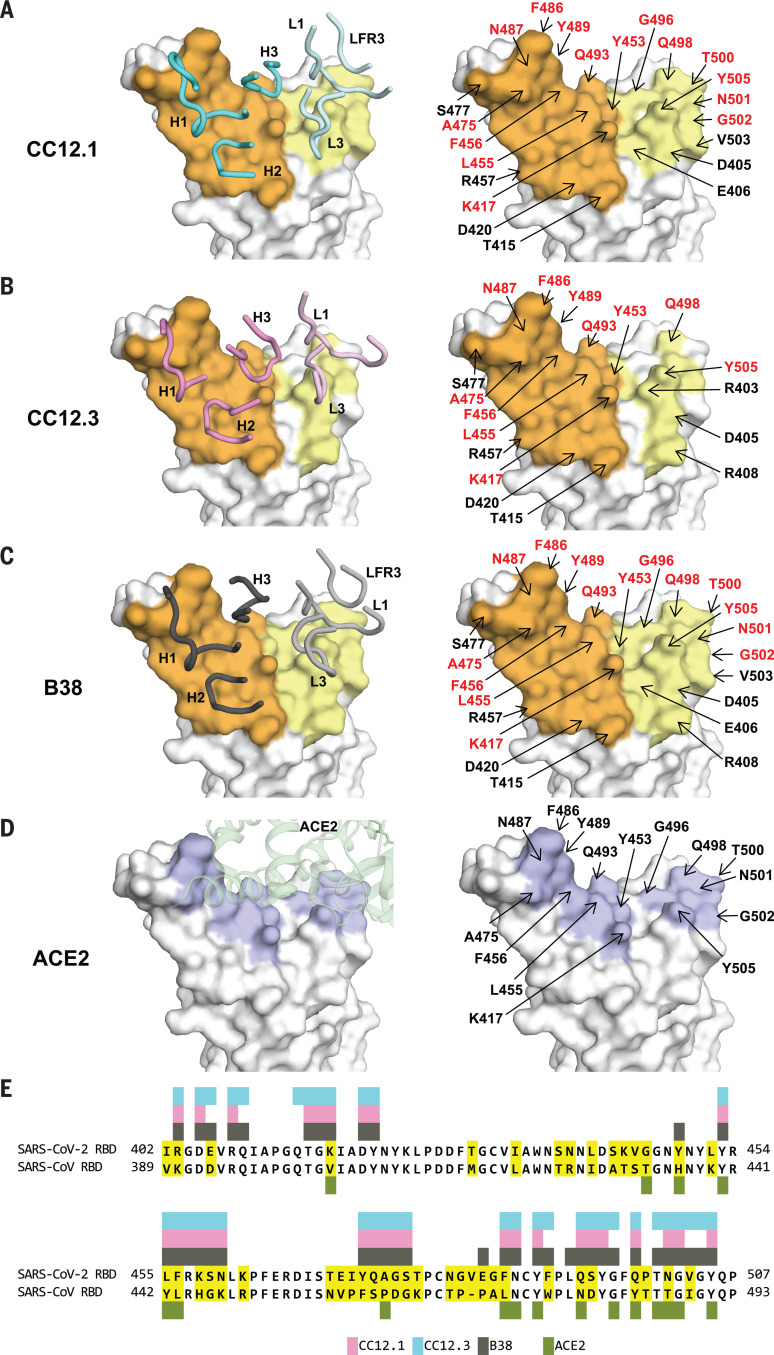
Epitopes of IGHV3-53 antibodies. (**A** to **C**) Epitopes of (A) CC12.1, (B) CC12.3, and (C) B38 (PDB 7BZ5) (*23*). Epitope residues contacting the heavy chain are shown in orange and those contacting the light chain are shown in yellow. CDR loops are labeled in the left panels; epitope residues are labeled in the right panels. For clarity, only representative epitope residues are labeled. Epitope residues that are also involved in ACE2 binding are shown in red. (**D**) ACE2-binding residues are shown in blue. ACE2 is shown in green in the left panel in a semitransparent cartoon representation. ACE2-binding residues are labeled in the right panel. A total of 17 residues were used for ACE2 binding (*12*), but only 15 are labeled here because the other two are at the back of the structure in this view and do not interact with the antibodies here. (**E**) Epitope residues for CC12.1, CC12.3, and B38 were identified by PISA (*41*) and annotated on the SARS-CoV-2 RBD sequence, which is aligned to the SARS-CoV RBD sequence with nonconserved residues highlighted. The 17 ACE2-binding residues were identified from a SARS-CoV-2 RBD–ACE2 complex structure as described previously (*12*).

We determined four complex crystal structures, CC12.1/RBD, CC12.3/RBD, CC12.1/RBD/CR3022, and CC12.3/RBD/CR3022, at resolutions of 3.20, 2.33, 2.70, and 2.90 Å, respectively (table S2). CC12.1 and CC12.3 bind to the ACE2-binding site on SARS-CoV-2 RBD with an identical angle of approach (Fig. 1, B to D, and fig. S5). Another IGHV3-53 antibody, B38, the structure of which was determined recently (*23*), binds to the ACE2-binding site on SARS-CoV-2 RBD in a similar manner but with a *K*_d_ of 70.1 nM (fig. S6). Similar to the ACE2-binding site (*11*), the epitopes of these antibodies can only be accessed when the RBD is in the “up” conformation (fig. S7). Among 17 ACE2-binding residues on RBD, 15 and 16 are within the epitopes of CC12.1 and B38, respectively, and 11 are in the epitope of CC12.3 (Fig. 2, A to D). Many of the epitope residues are not conserved between SARS-CoV-2 and SARS-CoV (Fig. 2E), explaining their lack of cross-reactivity (*27*). The buried surface area (BSA) from the heavy-chain interaction is quite similar in CC12.1 (723 Å^2^), CC12.3 (698 Å^2^), and B38 (713 Å^2^). By contrast, the light-chain interaction is much smaller for CC12.3 (176 Å^2^) compared with CC12.1 (566 Å^2^) and B38 (495 Å^2^), consistent with different light-chain gene usage. Although both CC12.1 and B38 use IGKV1-9, CC12.3 uses IGKV3-20, which suggests that IGHV3-53 can pair with different light chains to target the ACE2-binding site of the SARS-CoV-2 RBD. CC12.1 (56% BSA from the heavy chain) binds the RBD with similar affinity to CC12.3 (80% BSA from the heavy chain) but with a slightly slower dissociation rate (fig. S3), which might be influenced by the different light chain and its greater contribution in CC12.1. Nevertheless, the light-chain identity seems not to be as critical as the heavy chain. In fact, among the RBD-targeting IGHV3-53 antibodies, nine different light chains are observed, although IGKV1-9 and IGKV3-20 are the most frequently found to date (fig. S8).

**Fig. 3:**
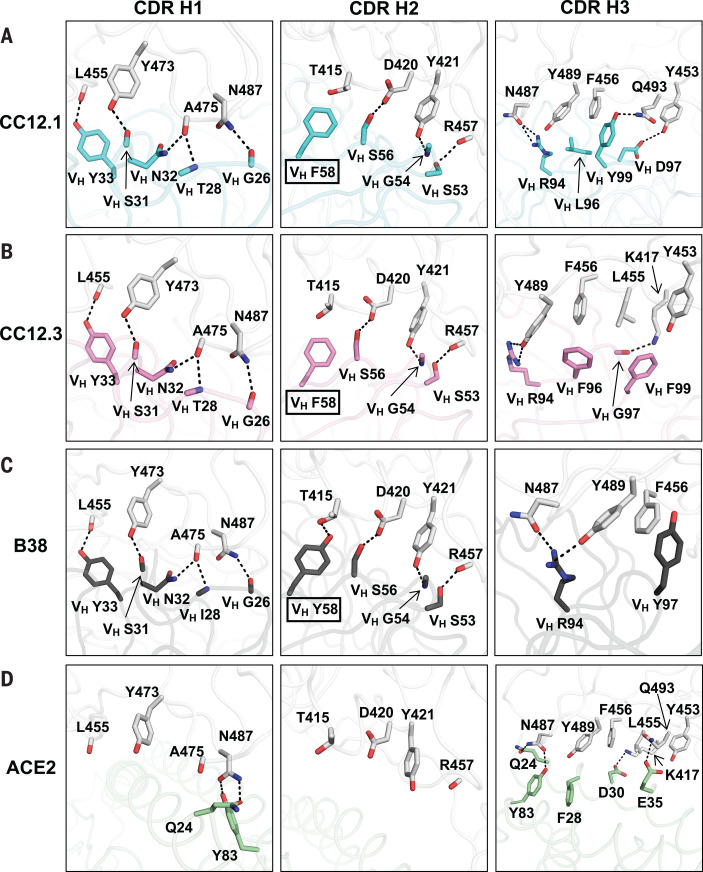
Interactions between the RBD and heavy-chain CDR loops. (**A** to **C**) Highly similar interaction modes between SARS-CoV-2 RBD and the antibody CDR H1 and H2 loops, but not the H3 loop, are observed for (A) CC12.1, (B) CC12.3, and (C) B38 (PDB 7BZ5) (*23*). The RBD is shown in white and antibody residues in cyan, pink, and dark gray, respectively. Oxygen atoms are shown in red and nitrogen atoms in blue. Hydrogen bonds are represented by dashed lines. (**D**) Interaction between ACE2 (green) and residues of the RBD (PDB 6M0J) (*12*) shown in (A) to (C).

To understand why IGHV3-53 is elicited as a shared antibody response, the molecular interactions between the RBD and the heavy chains of CC12.1, CC12.3, and B38 were analyzed. The complementarity-determining regions (CDRs) H1 and H2 of these antibodies interact extensively with the RBD mainly through specific hydrogen bonds (Fig. 3, A and B). All residues on CDR H1 and H2 that hydrogen bond with the RBD are encoded by the germ line IGHV3-53 (fig. S2 and table S3). These interactions are almost identical among CC12.1, CC12.3, and B38, with the only difference at the variable region of immunoglobulin heavy chain (V_H_) residue 58. A somatic mutation V_H_ Y58F in CC12.1 and CC12.3, but not in B38 (Fig. 3, A to C, boxed residues, and fig. S9), results in similar van der Waals interactions, with only a loss of a single hydrogen bond from the hydroxyl of the germ line Tyr in B38 to the RBD (Fig. 3C). None of these antibody interactions mimics ACE2 binding (Fig. 3D).

**Fig. 4:**
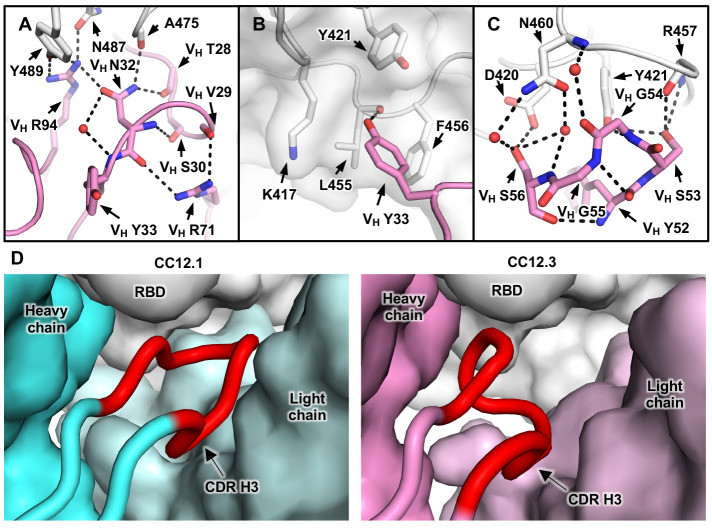
Two IGHV3-53 germline-encoded motifs with a short CDR H3. (**A**) Illustration of the extensive hydrogen bond network that involves V_H_ N32 of the NY motif in CDR H1. (**B**) Hydrophobic cage interaction between the RBD and V_H_ Y33 of the NY motif in CDR H1. (**C**) Hydrogen bond network that involves the SGGS motif in CDR H2. CC12.3 is shown here because its structure is at higher resolution than CC12.1. (**D**) CDR H3 length is constrained to fit in a relatively small pocket on the RBD surface. The heavy and light chains of CC12.1 (cyan) and the RBD (white) are shown in surface representation, with CDR H3 (red) highlighted in the diagram in the left panel. CC12.3 (pink) is shown in the right panel in the same representation.

Our structural analysis reveals two key motifs in the IGHV3-53 germline sequence that are important for RBD binding: an NY motif at V_H_ residues 32 and 33 in the CDR H1 and an SGGS motif at V_H_ residues 53 to 56 in the CDR H2 (Fig. 3 and fig. S10). The side chain of V_H_ N32 in the NY motif hydrogen bonds with the backbone carbonyl of A475 on the RBD, and this interaction is stabilized by an extensive network of hydrogen bonds with other antibody residues as well as a bound water molecule (Fig. 4A). V_H_ N32 also hydrogen bonds with V_H_ R94, which in turn hydrogen bonds with N487 and Y489 on the RBD (Fig. 4A). These polar contacts not only enhance the RBD-Fab interaction but also stabilize the CDR conformations with the surrounding residues (framework). V_H_ Y33 in the NY motif inserts into a hydrophobic cage formed by RBD residues Y421, F456, and L455 and the aliphatic component of K417 (Fig. 4B). A hydrogen bond between V_H_ Y33 and the carbonyl oxygen of L455 on the RBD further strengthens the interaction. The second key motif, SGGS, in CDR H2 forms an extensive hydrogen bond network with the RBD (Fig. 4C), including four hydrogen bonds that involve the hydroxyl side chains of V_H_ S53 and V_H_ S56 and four water-mediated hydrogen bonds to the backbone carbonyl of V_H_ G54, the backbone amide of V_H_ S56, and the side chain of V_H_ S56. Along with V_H_ Y52, the SGGS motif takes part in a type I beta turn, with a positive Φ angle for V_H_ G55 at the end of the turn. In addition, the Cα of V_H_ G54 is only 4 Å away from the RBD, indicating that side chains of other amino acids would clash with the RBD if they were present at this position.

The NY and SGGS motifs, important for RBD binding, are both encoded in the IGHV3-53 germline gene. In addition to IGHV3-53, only the closely related IGHV3-66 contains an NY motif in CDR H1 and an SGGS motif in CDR H2 (*33*). IGHV3-66 is commonly observed (*19*–*22*, *24*, *26*) in antibodies in SARS-CoV-2 patients (Fig. 1A) and is also well represented in the repertoire of healthy individuals (0.3 to 1.7% of total antibodies) (*29*). Overall, our structural analysis has identified two germline-encoded binding motifs that enable IGHV3-53 to target the SARS-CoV-2 RBD, with mutations apparently not required from affinity maturation.

Although the binding mode of CDR H1 and H2 to RBD is highly similar among CC12.1, CC12.3, and B38, the interaction of CDR H3 with the RBD varies (Fig. 3, A to C) because of differences in the CDR H3 sequences and conformations (fig. S1 and Fig. 4D). For example, whereas CDR H3 of CC12.1 interacts with RBD Y453 through a hydrogen bond, CDR H3 of CC12.3 and B38 do not form such a bond (Fig. 3, A to C). Similarly, because of the difference in light-chain gene usage, light-chain interactions with the RBD can vary substantially in IGHV3-53 antibodies (fig. S11). Overall, our structural analysis demonstrates that IGHV3-53 provides a versatile framework with which to target the ACE2-binding site in SARS-CoV-2 RBD.

An interesting feature of CC12.1 and CC12.3 is their relatively short CDR H3. Although the CDR H3 sequences of CC12.1 and CC12.3 differ, both are nine amino acids in length (Kabat numbering) (*34*). Antibody B38 has an even shorter CDR H3 of seven residues (*23*). The average CDR H3 length for human antibodies is ~13 (*35*), although very long CDR H3s (up to 30 residues or more) are found in many broadly neutralizing antibodies to HIV-1 (*36*). Longer CDR H3s cannot be accommodated in these IGHV3-53 antibodies because their epitopes are relatively flat, with only a small pocket to insert their CDR H3 loop (Fig. 4D). A similar conclusion was reached in another recent study, which also reported that SARS-CoV-2 RBD-targeting antibodies encoded by IGHV3-53 or IGHV3-66 tend to have a short CDR H3 (*28*, *37*). Among the RBD-targeting antibodies reported recently (*17*–*28*), IGHV3-53 antibodies consistently have a significantly shorter CDR H3 compared with those encoded by other IGHV genes (*P* = 6e-8, Mann-Whitney *U* test) (fig. S12) and are also shorter than IGHV3-53–encoded antibodies in the naïve human antibody repertoire (*30*). Thus, a short CDR H3 length is one of the molecular features of the IGHV3-53–encoded antibody response to SARS-CoV-2 RBD, reminiscent of a below-average five-residue CDR L3 in IGHV1-2 antibodies to the receptor-binding site in HIV-1 Env gp120 (*38*). Nevertheless, a small subset of these IGHV3-53 antibodies have longer CDR H3s that warrant further investigation of their binding mode (fig. S12).

In addition to IGHV3-53, several other IGHV genes, such as IGHV1-2, IGHV3-9, and IGHV3-30, are also more frequently observed than other germ lines in SARS-CoV-2 RBD-targeting antibodies (Fig. 1A). Future work will investigate the molecular mechanisms of these IGHV responses to SARS-CoV-2, as well as whether other germline gene segments, including IGHD and the light chain, contribute in recurring motifs to the SARS-CoV-2 antibody response. The characterization of these IGHV3-53 antibodies to SARS-CoV-2 is a promising starting point for rational vaccine design (*39*), given that limited to no affinity maturation is required to achieve a highly potent neutralizing antibody response to the RBD. Because IGHV3-53 is found at a reasonable frequency in healthy individuals (*29*, *30*), this particular antibody response could be commonly elicited during vaccination (*40*).

## SUPPLEMENTARY MATERIALS

science.sciencemag.org/content/369/6507/1119/suppl/DC1 Materials and Methods Figs. S1 to S12 Tables S1 to S3 References (*42*–*50*) MDAR Reproducibility Checklist

## Appendix

View/request a protocol for this paper from *Bio-protocol*.
